# Can C-reactive Protein Increase the Efficiency of the Bedside Index of Severity in Acute Pancreatitis Scoring System?

**DOI:** 10.7759/cureus.4205

**Published:** 2019-03-07

**Authors:** Yavuz Yigit, Kübra Selçok

**Affiliations:** 1 Emergency Medicine, University of Health Sciences, Kocaeli Derince Training and Research Hospital, Kocaeli, TUR

**Keywords:** bisap, crp=c-reactive protein, acute pancreatitis, pancreatic inflammation

## Abstract

Background

Early diagnosis and accurate assessment of the severity of the disease are critical factors in the management of acute pancreatitis (AP). In this study, we investigated the success rates of combinations of Bedside Index of Severity in Acute Pancreatitis (BISAP) scores with C-reactive protein (CRP) values in predicting severe AP.

Methods

The medical records of all patients with AP admitted to our hospitals from September 2015 to September 2018 were reviewed retrospectively. To evaluate the severity of AP, the revised Atlanta criteria were used, and patients who developed organ failure lasting more than 48 hours were considered to have severe AP. We analyzed patient CRP values at the 24-hour mark via receiver operating characteristic (ROC) curve analysis. Four groups were then formed to separate mild AP from moderate to severe AP. The first group had BISAP scores ≥ 3, the second group had CRP values ≥ 90.7 mg/L, the third group had BISAP scores ≥ 3 and CRP values ≥ 90.7 mg/L, and the fourth group had BISAP scores ≥ 3 or measured CRP values ≥ 90.7 mg/L. Predictive accuracy, sensitivity, specificity, and positive and negative predictive values of groups in the prediction of severe AP were calculated.

Results

Our study population consisted of 207 patients, and according to the revised Atlanta scoring, 165 patients (79.7%) had mild AP, 30 (14.4%) had moderate, and 12 (5.8%) had severe AP. Comparing the mild, moderate, severe AP groups, we noted a significant difference between the mean hospital stay time, BISAP scores, and CRP values (p<0.001). Group 1, 2, 3, and 4 values of mild AP and all severe AP (moderate and severe) were significant (p<0.001). The highest specificity values were found in Group 3 (97.6%), while the highest sensitivity values were observed in Group 4 (88.1%).

Conclusion

CRP may increase the success of BISAP scoring in predicting the severity of AP.

## Introduction

Acute pancreatitis (AP) is an inflammatory condition which may be mild or severe; in severe cases, pancreatic enzymes can cause damage to the gland itself [1]. AP has many different etiologies, and overall mortality is 5% to 10%. Most cases (80% to 90%) are mild or self-limited and have a good prognosis. The remaining 10% to 20% of cases warrant monitoring in intensive care units due to pancreatic necrosis or distant organ damage. Severe AP cases usually require surgical intervention, and overall mortality can be up to 40% [2].

Early diagnosis and accurate assessment of the severity of the disease are very important factors in the initial evaluation and management of the disease. Mild cases can be managed by fluid resuscitation and supportive treatment, but severe cases usually require nutritional support and intensive care follow-up. It is crucial for clinicians to identify these cases because severe cases may deteriorate rapidly [3].

Some scoring systems based on clinical and biochemical data have been used for the last 50 years, including Ranson’s Criteria for Pancreatitis Mortality, first described in 1974, the Bedside Index of Severity in Acute Pancreatitis (BISAP), and the Acute Physiology and Chronic Health Evaluation (APACHE) II. Each of these scoring systems has its own limitations, such as low sensitivity and specificity, the complexity of the scoring system, and the inability to achieve a final score up to 48 hours after admission [4].

Ranson’s Criteria, as a scoring system, was a major step in evaluating the severity of the disease and has been involved in clinical use for over 40 years. Ranson’s criteria is moderately successful in evaluating disease severity. Obtaining the score requires a very valuable period of 48 hours (especially valuable regarding early treatment) [5]. The Ranson scoring system also includes parameters not routinely used in most hospitals.

BISAP is a scoring system where the severity of the disease can be evaluated during admission. BISAP contains five parameters: blood urea nitrogen (BUN) > 25 mg/dL, mental state deterioration, age > 60 years, pleural effusion, and ≥ 2 systemic inflammatory response syndrome criteria. The mean mortality for each positive parameter increases, for example, when the score is 0, the mortality is 0.20%, and the mortality rate is 22% to 27% if all parameters are positive [6]. BISAP has been validated in many prospective cohort studies and has proven useful in clinical follow-up to predict necrosis and mortality. The biggest advantage of Ranson’s criteria compared to BISAP is that Ranson’s informs on the development of persistent organ failure, BISAP does not provide information about temporary or persistent organ failure. To calculate BISAP, we need anamnesis, physical examination, simple laboratory tests, and chest radiography, and all of these parameters are evaluated in routine emergency department processes. The superiority of BISAP is due to its simplicity and the fact that the patient can be easily calculated during emergency service follow-up [7]. However, publications are reporting the sensitivity of BISAP for AP severity as 37.5% [8] and specificity as 50% [9]. Therefore, there is a need for a parameter to be evaluated in the practical, emergency service routine which will contribute to the sensitivity and specificity of the BISAP.

C-reactive protein (CRP) is still one of the more useful biochemical parameters, although it still reaches its peak at 72 hours after the onset of symptoms. However, as far as we know, there is still no published study on the success of BISAP in combination with CRP regarding predicting the severity of pancreatitis [10].

The aim of this study was to investigate the success rates of combinations of BISAP with CRP values in predicting severe AP.

## Materials and methods

We scanned the electronic data system and gathered files of patients hospitalized from September 2015 to September 2018 with the diagnosis of AP in the emergency room of our hospital. Patients with incomplete files or electronic records, acute cholecystitis or cholangitis during admission or follow-up, or chronic pancreatitis in previous computed tomography (CT) images or in hospitalization were excluded from our study. We also excluded patients who did not have biliary ultrasound imaging in the first 24 hours after admission, patients from another center, patients diagnosed with AP outside of our emergency department, and patients whose CRP levels were studied at the 24th hour of admission. The patients who were admitted to our hospital with the diagnosis of AP in our hospital according to the revised 2012 Atlanta criteria (i.e., abdominal pain suggestive of AP, increase in amylase and/or lipase levels three times higher than normal, and CT findings in classic AP findings) [11] were included in the study. To evaluate the severity of AP, the revised Atlanta criteria were used, and patients who developed organ failure lasting more than 48 hours were considered to have severe AP. The cases with local or systemic complications and/or in whom organ failure was detected in under 48 hours were accepted as moderate or severe pancreatitis. The others were considered as mild AP. Organ failure in three organ systems (i.e., renal, respiratory and cardiovascular) were examined according to the modified Marshall score [11].

CRP values of the patients who were hospitalized with the diagnosis of AP at the 24th hour were examined by receiver operating characteristic (ROC) curve analysis. The CRP cut-off value with the highest sensitivity and specificity was found in the differentiation of mild and moderate or severe AP, and this value was noted as the optimal CRP value. Four groups then formed to separate mild AP from moderate to severe AP. Cut-off values were BISAP ≥ 3 for the first group, CRP ≥ optimal CRP value measured for the second group, BISAP ≥ 3 and measured CRP ≥ optimal CRP for the third group, BISAP ≥ 3 or measured CRP ≥ optimal CRP for the fourth group. We compared the sensitivity and specificity of these four groups in the evaluation of mild AP with that of severe AP.

In our study, statistical calculations were performed using the IBM Statistical Package for the Social Sciences 22 (IBM Corp., Armonk, NY, USA) program. Normally distributed continuous variables were expressed as mean standard deviation. The median values of other continuous variables were shared. Frequency and percentage values of categorical variables were shared. Within the scope of our study, CRP values which were examined at 24th hours of hospitalization were examined by ROC analysis and CRP cut-off value with optimal sensitivity and specificity was found to distinguish mild acute pancreatitis from moderate and severe acute pancreatitis and this value is called optimal CRP. Four groups were then formed to separate mild acute pancreatitis from moderate to severe acute pancreatitis. Cut-off values were listed as BISAP ≥ 3 for the first group, CRP ≥ optimal CRP value measured for the second group, for the third group BISAP ≥ 3 and measured CRP ≥ optimal CRP, for the fourth group BISAP ≥ 3 or measured CRP ≥ optimal CRP. Afterwards, the sensitivity and specificity values of these four groups were determined in differentiating mild acute pancreatitis from severe acute pancreatitis. Chi-square or Fisher exact test was used to compare the groups.

## Results

The mean age of our patients was 58 ± 17 and female/male ratio was 1.1 (108/99). Biliary etiology was found in 62.8% (130) of the patients, idiopathic in 13.5% (28), hyperlipidemia in 7.7% (16), and alcoholism was found in 4.3% (9). According to the revised Atlanta criteria, 165 cases (79.7%) were mild, 30 (14.4%) were moderate, and 5.8 (12%) were severe pancreatitis, and when these three groups were compared, we found a significant difference between the mean hospital stay time, BISAP scores, and CRP values (p <0.001). Group 1, 2, 3, and 4 of mild, moderate, and severe AP were significantly different from each other (p <0.001) (Table 1).

**Table 1:** Patient characteristics, BISAP score and CRP value, and AP severity AP, acute pancreatitis; BISAP, Bedside Index of Severity in Acute Pancreatitis; CRP, C-reactive protein; IQR, interquartile range; SD, standard deviation.

	Mild pancreatitis (n=165)	Moderate pancreatitis (n=30)	Severe pancreatitis (n=12)	p
Female/Male Ratio	86/79 1.08	16/14 1.14	6/6 1	0.981
Age ± SD	57 ± 17	60 ± 18	68 ± 16	0.084
Mean inpatient time (IQR)	5 (4-7)	7 (5-10.25)	11 (6.25-31.25)	0.001
BISAP score (IQR)	1 (0-2)	3 (2-4)	4 (3.25-5)	<0.001
CRP value (mg/L, IQR)	13.6 (2.6-52.4)	104.5 (95-111.25)	162.75 (53.45-243.15)	<0.001
Group 1 (BISAP ≥ 3), n(%)	17 (10.3)	17 (56.7)	10 (83.3)	<0.001
Group 2 (CRP ≥ 90.7 mg/L), n(%)	21(12.7)	25(83.3)	9 (75)	<0.001
Group 3 (BISAP ≥ 3 and CRP ≥ 90.7), n (%)	4 (2.4)	16 (53.3)	8 (66.7)	<0.001
Group 4 (BISAP ≥ 3 or CRP ≥ 90.7), n (%)	34 (20.6)	26 (86.7)	11 (91.7)	<0.001

Results were significantly different when Group 1, 2, 3, and 4 values of mild pancreatitis were compared separately with those with moderate and severe pancreatitis (p <0.001). Eight patients (3.8%) died due to AP. There was also a significant difference between the survivors and the patients who died in Groups 1, 2, 3, and 4 (p <0.001) (Tables 2-4).

**Table 2:** Groups’ sensitivity, specificity, negative and positive predictive values and overall accuracy to detect moderately severe AP AP, acute pancreatitis; BISAP, Bedside Index of Severity in Acute Pancreatitis; CI, confidence interval; CRP, C-reactive protein; PPV, positive predictive value; NPV, negative predictive value; ACC, accuracy.

	Moderate pancreatitis (n=30) n (%)	Mild pancreatitis (n=165) n (%)	p	Odds ratio (CI 95%)	Sensitivity %	Specificity %	PPV %	NPV %	ACC %
Group 1 (BISAP ≥ 3)	17 (56.7)	17 (10.3)	<0.001	11.3 (4.7-27.4)	56.7	89.7	50	91.9	84.6
Group 2 (CRP ≥ 90.7 mg/L)	25 (83.3)	21 (12.7)	<0.001	34.2 (11.8-99.3)	83.3	87.3	54.3	96.6	86.6
Group 3 (BISAP ≥ 3 and CRP ≥ 90.7 mg/L)	16 (53.3)	4 (2.4)	<0.001	46 (13.5-156.4)	53.3	97.6	80	92	90.7
Group 4 (BISAP ≥ 3 or CRP ≥ 90.7 mg/L)	26 (86.7)	34 (20.6)	<0.001	25 (8.1-76.6)	86.7	79.4	43.3	97	80.5

**Table 3:** Groups’ sensitivity, specificity, negative and positive predictive values and overall accuracy to detect severe AP AP, acute pancreatitis; BISAP, Bedside Index of Severity in Acute Pancreatitis; CI, confidence interval; CRP, C-reactive protein; PPV, positive predictive value; NPV, negative predictive value; ACC, accuracy.

	Severe pancreatitis (n=12) n (%)	Mild pancreatitis (n=165) n (%)	p	Odds ratio (CI 95%)	Sensitivity %	Specificity %	PPV %	NPV %	ACC %
Group 1 (BISAP ≥ 3)	10 (83.3)	17 (10.3)	<0.001	43.5 (8.7-215.3)	83.3	89.7	37	98.7	89.2
Group 2 (CRP ≥ 90.7 mg/L)	9 (75)	21 (12.7)	<0.001	20.5 (5.1-82.1)	75	87.3	30	98	86.4
Group 3 (BISAP ≥ 3 and CRP ≥ 90.7 mg/L)	8 (66.7)	4 (2.4)	<0.001	80.5 (16.9-382)	66.7	97.6	66.7	97.6	95.4
Grup 4 (BISAP ≥ 3 or CRP ≥ 90.7 mg/L)	11 (91.7)	34 (20.6)	<0.001	42.3 (5.2-339.7)	91.7	79.4	24.4	99.2	80.2

**Table 4:** Groups’ sensitivity, specificity, negative and positive predictive values and overall accuracy to detect mortality AP, acute pancreatitis; BISAP, Bedside Index of Severity in Acute Pancreatitis; CI, confidence interval; CRP, C-reactive protein; PPV, positive predictive value; NPV, negative predictive value; ACC, accuracy.

	Died (n=8) n (%)	Survivors (n=199) n (%)	p	Odds ratio (CI 95%)	Sensitivity %	Specificity %	PPV %	NPV %	ACC %
Group 1 (BISAP ≥ 3)	8 (100)	36 (18.1)	<0.001	5.5 (4.1-7.4)	100	81.9	18.2	100	82.6
Group 2 (CRP ≥ 90.7 mg/L)	6 (75)	49 (24.6)	0.002	3 (1.9-4.8)	75	75.4	10.9	98.7	73.9
Group 3 (BISAP ≥ 3 and CRP ≥ 90.7 mg/L)	6 (75)	22 (11.1)	<0.001	6.7 (3.8-11.8)	75	88.9	21.4	98.9	88.4
Grup 4 (BISAP ≥ 3 or CRP ≥ 90.7 mg/L)	8 (100)	63 (31.7)	<0.001	3.1 (2.5-3.8)	100	69.3	11.3	100	69.5

Group 1, 2, 3, and 4 values of mild AP and all severe AP cases (moderate and severe) were significant (p <0.001). The highest specificity values were found in Group 3 (97.6%), while the highest sensitivity values were observed in Group 4 (88.1%) (Table 5).

**Table 5:** Groups’ sensitivity, specificity, negative and positive predictive values and overall accuracy to detect mild AP AP, acute pancreatitis; BISAP, Bedside Index of Severity in Acute Pancreatitis; CI, confidence interval; CRP, C-reactive protein; PPV, positive predictive value; NPV, negative predictive value; ACC, accuracy.

	All severe pancreatitis (moderate and severe) (n=42) n (%)	Mild Pancreatitis (n=165) n (%)	p	Odds ratio (CI 95%)	Sensitivity %	Specificity %	PPV %	NPV %	ACC %
Group 1 (BISAP ≥ 3)	27 (64.3)	17 (10.3)	<0.001	15.6 (6.9-35.1)	64.3	89.7	61.4	90.8	84.5
Group 2 (CRP ≥ 90.7 mg/L)	34 (81)	21 (12.7)	<0.001	29.1 (11.9-71.4)	81	87.3	81	94.7	86.8
Group 3 (BISAP ≥ 3 and CRP ≥ 90.7 mg/L)	24 (57.1)	4 (2.4)	<0.001	53.6 (16.7-172)	57.1	97.6	85.7	89.9	89.3
Grup 4 (BISAP ≥ 3 or CRP ≥ 90.7 mg/L)	37 (88.1)	34 (20.6)	<0.001	28.5 (10.4-78.1)	88.1	79.4	52.1	96.3	74

**Figure 1:**
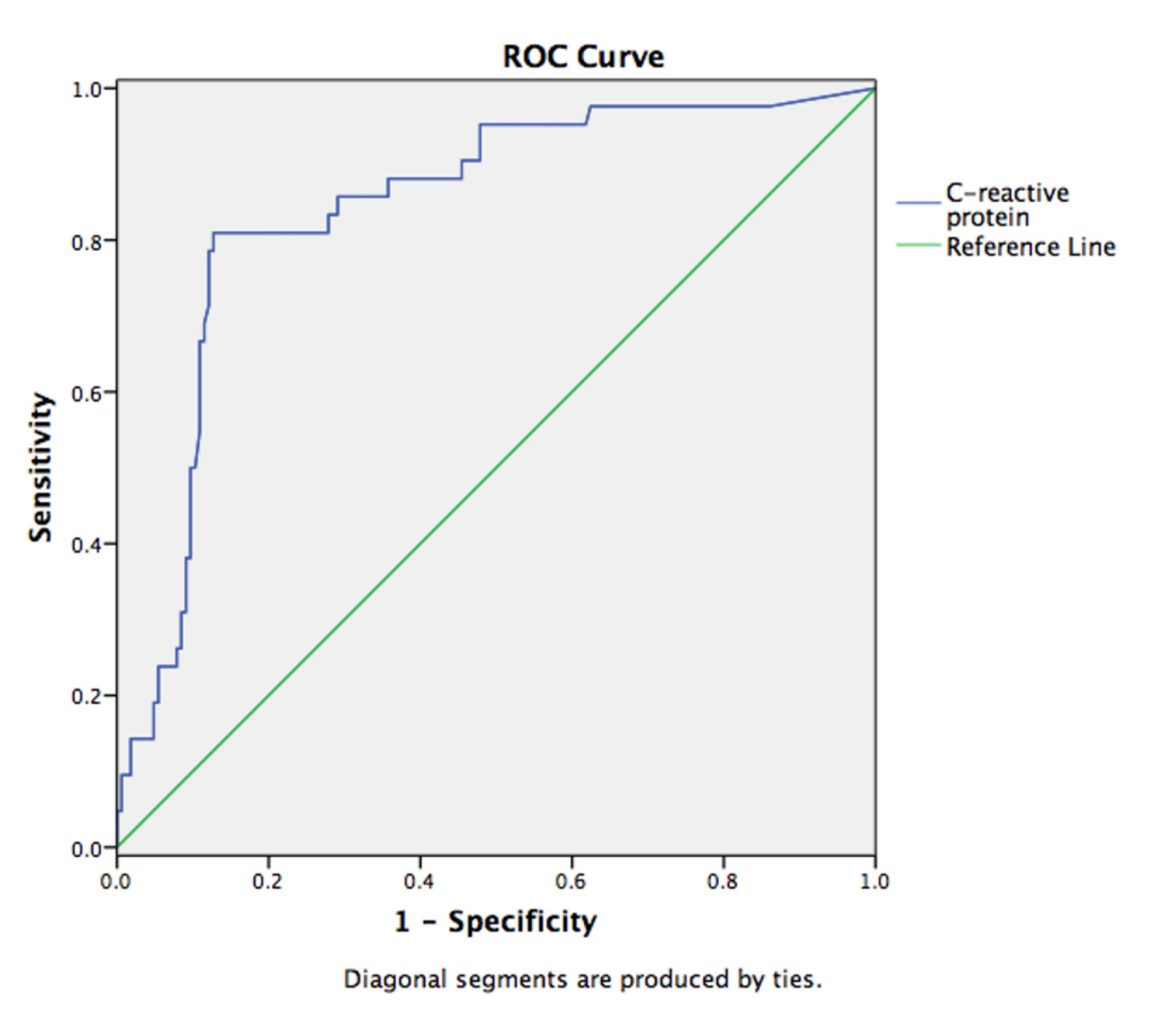
ROC analysis with a chosen cutoff value of CRP ≥ 90.7 mg/L to detect mild acute pancreatitis ROC, receiver operating characteristic; CRP, C-reactive protein.

We used ROC analysis and chose a cutoff value of CRP ≥ 90.7 mg/L with a sensitivity and specificity of 81% and 87.3%, respectively (area under the curve, 0.842; p =0.001; confidence interval, 0.776 to 0.908 (Figure 1).

## Discussion

The combination of CRP values with BISAP values had high sensitivity and specificity for predicting the severity of pancreatitis. In the recent literature, severe AP rate has a 10% to 20% incidence rate, and the mortality rate for severe AP is 20% to 30% [3,11-12]. Using the last Atlanta classification, the rate of severe AP was 20.1%, and the mortality rate in this group was 28.5%. Carnovale et al. reported an overall mortality rate of 4.8% [12], and Singh et al. reported an overall mortality rate of 3.5% [13]. Similarly, 3.8% of the patients died in our study [6,13]. BISAP is a simple and practical scoring system that leads to significant changes in the management of patients or predicts intensive care needs [6,14]. In a recent meta-analysis of 1,972 patients, sensitivity and specificity values were 64.82% and 83.62%, respectively, in predicting severe AP [15]. In another meta-analysis of 38,985 patients from four different countries, sensitivity values were 51% (43% to 60%) and specificity was 91% (89% to 92%) for severe AP [16]. BISAP results have similar sensitivity and specificity to the literature for predicting severe pancreatitis.

CRP is an acute phase protein produced by hepatocytes upon stimulation by cytokines interleukin-6, tumor-necrosis factor-alpha, and interleukin-1 during the acute phase response. It is an objective indicator of inflammation, and CRP levels have been shown to correlate well with clinical disease activity in gastrointestinal diseases such as Crohn's disease and AP [17]. Many researchers have investigated the success of CRP in predicting the severity of AP, and they examined the values measured at the time of admission to the hospital and 24, 48 or 72 hours after the admission and used cut-off values ranging from 110 mg/L to 150 mg/L. In these studies, the sensitivity, specificity, and positive and negative predictive values at the time of application were 38% to 61%, 89% to 90%, 59% to 78%, and 78% to 79%. The values at 24 hours were 44% to 83%, 70% to 96%, 42% to 89%, and 76% to 91%. The 48-hour values were 57% to 89%, 55% to 82%, 37% to 73%, and 80% to 94%. The 72-hour values were 83% to 90%, 60% to 84%, 75% to 86%, and 69% to 92% [18].

The sensitivity values in our study were slightly above those found in the literature due to our study’s lower CRP cut-off value. The high sensitivity values we found were particularly important because the most important problem of BISAP in terms of predicting the severity of AP was the low sensitivity values. However, in Group 4, BISAP and CRP values were evaluated jointly, and sensitivity values reached 88%.

Predicting the severity of AP is still a challenge. Although many systems have been developed for early detection of severity and prognosis of the disease, none of these systems are excellent [19]. Many models have been defined based on clinical, radiological, and laboratory findings. Some clinical indicators were used for this purpose such as advanced age (> 75 years), but obesity and alcoholic pancreatitis poor prognostic indicators [20-21].

Studies that use the clinical judgment of experienced physicians had sensitivity and specificity values of 39% and 93%, respectively [22]. Most clinical scoring systems are not suitable for use by emergency physicians [6,23-24]. For example, the most well-known scoring system, Ranson’s, is not suitable for use in the emergency department because it requires at least 48 hours of hospitalization to complete [23]. Furthermore, much of Ranson’s criteria evaluations cannot be conducted in the emergency department.

Another scoring system used in this field is the Glasgow-Imrie Criteria, which is similar to Ranson’s Criteria and has the same feasibility problems in the emergency department [23]. The APACHE II clinical scoring system can help in predicting the severity of pancreatitis, and studies report the 48th-hour values are more successful than the Ranson’s and Glasgow-Imrie Criteria, however, the complexity of the APACHE II scoring system is not suitable for use in the emergency department [25]. Therefore, a simple, easy to apply marker is needed with high sensitivity and specificity values feasible for use in the emergency department. The combination of BISAP and CRP provides the solution.

Our study was limited by its retrospective design and conducted in a single centre with a relatively small sample size, which limits the generalisability of our findings.

## Conclusions

As a means of predicting the severity of AP, the use of CRP in combination with the BISAP scoring system is a promising option over existing prediction methods and is feasible for deployment in emergency department situations.
